# Supplementary material for: Follow the money: Investigating gender disparity in industry payments among senior academics and leaders in plastic surgery
Source: PLoS One. 2020 Dec 28;15(12):e0235058. doi: 10.1371/journal.pone.0235058 (PMC7769471; doi:10.1371/journal.pone.0235058)
Supplement: S1 Table — (DOCX) [file pone.0235058.s001.docx]

| **S1 Table.** List of departmental leadership designations within each category | | |
| --- | --- | --- |
| **Chairs and Chiefs** | **Program Directors** | **Subdivision Heads** |
| Department Chair  Division Chief  Vice-Chair | Associate Program Director  Fellowship Director  Residency Program Director | Chief of Subspecialty Section  Clerkship Director  Clinical Director  Curriculum Director  Education Director  Head of Resident/Academic Affairs  Research or Laboratory Director |
