# Supplementary material for: Follow the money: Investigating gender disparity in industry payments among senior academics and leaders in plastic surgery
Source: PLoS One. 2020 Dec 28;15(12):e0235058. doi: 10.1371/journal.pone.0235058 (PMC7769471; doi:10.1371/journal.pone.0235058)
Supplement: S2 Table — (DOCX) [file pone.0235058.s002.docx]

| **S2 Table.** Definitions of Center for Medicare and Medicaid Services payment types. | |
| --- | --- |
| **Nature of payment** | **Definition** |
| Charitable contribution | Any payment or transfer of value made to an organization with tax-exempt status under the Internal Revenue Code of 1986, but only if it is not more specifically described by one of the other nature or payment categories. |
| Compensation for services other than consulting, including serving as faculty or as a speaker at an event other than a continuing education program. | Payments made to physicians for speaking, training, and education engagements that are not for continuing education. |
| Compensation for serving as faculty or as a speaker for an accredited or certified continuing education program | Compensation for serving as faculty or as a speaker for an accredited or certified continuing education program |
| Compensation for serving as faculty or as a speaker for an unaccredited and non-certified continuing education program | Compensation for serving as faculty or as a speaker for an unaccredited and non-certified continuing education program |
| Consulting fee | Payments made to physicians for advice and expertise on a particular medical product or treatment, typically provided under a written agreement and in response to a particular business need. These payments often vary depending on the experience of the physician being consulted. |
| Current or prospective ownership or investment interest | Ownership or investment interests currently held by physicians and teaching hospitals, as well as ownership interests or investments that physicians and teaching hospitals have not yet exercised. |
| Education | This category generally includes payments or transfers of value for classes, activities, programs or events that involve the imparting or acquiring of particular knowledge or skills, such as those used for a profession. This category can include things like textbooks and medical journal articles. |
| Entertainment | Attendance at recreational, cultural, sporting or other events that would generally have a cost. |
| Food and beverage | Food and beverage. |
| Gift | A general category, which will often include anything provided to a physician or teaching hospital that does not fit into another category. |
| Grant | Payments to a physician or teaching hospital in support of a specific cause or activity. |
| Honoraria | Similar to consulting fees, but generally reserved for a one-time, short duration activity. Also distinguishable in that they are generally provided for services which custom prohibits a price from being set. |
| Research | Payment for different types of research activities, including enrolling patients into studies of new drugs or devices. |
| Royalty or license | Royalty or other payment based on sales of products that use a physician’s intellectual property. |
| Space rental or facility fees | Fees for renting space or facilities, in a teaching hospital, for example. |
| Travel and lodging | Travel and lodging |
