# Supplementary material for: Follow the money: Investigating gender disparity in industry payments among senior academics and leaders in plastic surgery
Source: PLoS One. 2020 Dec 28;15(12):e0235058. doi: 10.1371/journal.pone.0235058 (PMC7769471; doi:10.1371/journal.pone.0235058)
Supplement: S3 Table — (DOCX) [file pone.0235058.s003.docx]

| **S3 Table.** Number of recipients and median dollar value of industry-sponsored speakerships to senior academic plastic surgeons and departmental leaders stratified by type of payment and gender. | | | |
| --- | --- | --- | --- |
| **Speakership subtype** | **Men (*n*=228)** | **Women (*n*=31)** | ***p*-value** |
| Event other than a continuing education program  Number of recipients  Median dollar value | 27 (12%)  $11,550 [IQR: 2,723 – 20,100] | 4 (13%)  $11,438 [IQR: 3,281 – 35,494] | 0.9998  - |
| Unaccredited and non-certified continuing education program  Number of recipients  Median dollar value | 1 (0.4%)  $32,753 | 0 (0%)  - | 0.9998  - |
| Accredited or certified continuing education program  Number of recipients  Median dollar value | 11 (5%)  $2,000 [IQR: 1,000 – 3,000] | 1 (3%)  $408 | 0.9998  - |
| *IQR* – interquartile range | | | |
