# Supplementary material for: Follow the money: Investigating gender disparity in industry payments among senior academics and leaders in plastic surgery
Source: PLoS One. 2020 Dec 28;15(12):e0235058. doi: 10.1371/journal.pone.0235058 (PMC7769471; doi:10.1371/journal.pone.0235058)
Supplement: Attachment — Submitted filename: Paper Review Comments WL.docx [file pone.0235058.s004.docx]

General comments:

The study applied a quite strict inclusion and exclusion criteria therefore results in a very limited sample size, especially including only 38 females. It’s unclear how representative is this study sample (as % of academic faculties). The merits of inclusion and exclusion criteria is also questionable. Faculty with the rank of associate professor maybe included, if not those with the rank of assistant professor. Those who processed no payment data for 2018 may also be included as it enables the examination of research question: what are the characteristics of academics who received industry payments.

Measurement of leadership position focuses only on departmental positions. This should be expanded to leadership position in professional societies as well as recipients of major rewards in plastic surgery.

Abstracts

1) Add description of inclusion and exclusion criteria
2) In Results paragraph line 39, change to “for speakerships where women received smaller amounts compared to their male counterparts (median payments of $3,675 vs. $7,134 for women and men respectively, P<0.0001)”.
3) In Results paragraph line 40-42, “Multiple linear regression … (R=0.17, … and R=0.14, …)” - Explain what is the R value.

Introduction

1) Lines 69-71, consider changing to “This paper aims to 1) characterize academics who received industry sponsorship; 2) examine relationship between industry sponsorship and gender, academic rank, leadership position, career length, and academic productivity”.

Method

1) Line 86, consider to include faculty with the academic rank of professor or associate professor.
2) Line 110, consider faculty with the academic rank of professor or associate professor or a leadership position.
3) Lines 112-113, consider deleting “(iv) who possessed no payment data for 2018” from the exclusion criteria.

Data analysis

1) Lines 124-125, explain the meaning of R statistic.

Results

1) Line 133, 316 differs from the sum of 279 and 38 in table 1.
2) Line 140, p=0.0024; Line 141, p=0.0096. Both p-values differ from the p-values in table 1.
3) Lines 147 and 150, re-organize figures: put 1a) and 2a), 1b) and 2b) together.
4) Lines 149-150, “This was also true when the groups were stratified by academic rank (Figure 1b) or leadership position (Figure 2b)”, this interpretation seems to be wrong. Differences between men and women appears to be large, especially in figure 2b) stratified by leadership position.
5) Lines 173, “After adjusting for confounding variables”, explain the list of confounding variables.

Tables

Table 1:

1) Report one p-value from the chi-square test examining the relationship between academic rank and gender, or leadership position and gender.
2) Report statistical tests used to obtain p-values for median H-index and median career length in footnote.

Table 2:

1) Report p-values comparing men and women on median dollar value for education and associated research.
